# Semaglutide and tirzepatide: Oral cavity effects of weight-loss therapies

**DOI:** 10.17179/excli2025-9065

**Published:** 2026-01-09

**Authors:** Maria Eduarda Bernardo, Felipe Gomes Dallepiane, Mario Escobar, Ariadne Cristiane Cabral Cruz, Cesar Augusto Magalhães Benfatti

**Affiliations:** 1 Post-Graduation Program of Dentistry, Center for Education and Research on Dental Implants, Federal University of Santa Catarina, Florianópolis, Brazil; 2 Department of Dentistry, Universidad Católica Santiago de Guayaquil (UCSG), Guayaquil, Ecuador; 3 Applied Virology Laboratory, Federal University of Santa Catarina, Florianópolis, Brazil

## ⁯

Medications such as Ozempic® (semaglutide) and Mounjaro® (tirzepatide), originally developed for the treatment of type 2 diabetes mellitus, belong to the class of incretin-based therapies and have demonstrated significant efficacy in both glycemic control and weight management (Ryan et al., 2024[6]). However, their popularity has expanded beyond medical indications, and they are now widely used for aesthetic purposes (Mawardi et al., 2023[4]; Sillassen et al., 2024[8]). This trend has resulted in indiscriminate use, often without proper medical supervision, raising concerns about systemic adverse events and, more specifically, their potential impact on oral health (Shu et al., 2022[7]).

The therapeutic action of glucacon-like peptide (GLP)-1 receptor agonists involves mimicking incretin activity, thereby stimulating insulin secretion in a glucose-dependent manner and suppressing glucagon release (Tan et al., 2022). In addition, they delay gastric emptying and act on the central nervous system to promote satiety and reduce caloric intake (Tan et al., 2022[9]; Sillassen et al., 2024[8]). Tirzepatide, unlike semaglutide, is a dual agonist targeting both gastric inhibitory polypeptide (GIP) and GLP-1 receptors, a mechanism associated with enhanced metabolic and weight loss benefits (Aronne et al., 2024[1]). Beyond glycemic effects, these medications also provide cardiovascular protection, including reduced blood pressure, lower incidence of hypertension, and anti-atherosclerotic activity (Sillassen et al., 2024[8]).

Despite its therapeutic benefits, semaglutide may exert notable adverse effects on the oral cavity. Xerostomia (dry mouth) is among the most frequently reported complications and is often accompanied by halitosis, colloquially referred to as “Ozempic breath” (Shu et al., 2022[7]; Bando et al., 2024[2]). Beyond discomfort, xerostomia compromises oral homeostasis by reducing the protective capacity of saliva, thereby increasing the risk of dental caries, demineralization, tooth sensitivity, candidiasis, and other oral diseases (Bando et al., 2024[2]). A recent case series described three patients who developed severe hyposalivation secondary to semaglutide therapy, underscoring a potential adverse effect with significant implications for quality of life (Mawardi et al., 2023[4]).

In some cases, patients treated with semaglutide also develop dysgeusia, a persistent sweet taste frequently referred to as “Ozempic tongue", which may arise within the first two weeks of therapy and has been documented in approximately 6 % of users (Volpe et al., 2023[10]). This phenomenon does not necessarily represent a negative or inadequate effect, but rather a taste alteration that may contribute to decreased appetite and weight reduction. Although considered an adverse event, it appears to have beneficial implications for metabolic control and glucose variability (Bando et al., 2024[2]). The detailed mechanism remains unclear; it has been hypothesized that semaglutide may alter genes related to taste receptors on the tongue, but no definitive explanation has been established, and further clarification is expected in future studies (Volpe et al., 2023[10]).

Gastrointestinal disturbances are also common adverse effects of both semaglutide and tirzepatide, particularly in the early stages of treatment, and include nausea, vomiting, diarrhea, and reflux (Wharton et al., 2022[11]). These conditions increase enamel exposure to acidity, contributing to dental erosion and dentin hypersensitivity (Chakraborty and Anjankar, 2022[3]). Recurrent vomiting episodes may further alter oral pH and disrupt the oral microbiome, aggravating the risk of mucosal inflammation and secondary infections (Xia et al., 2025[12]). Preventive guidance is therefore essential, including the use of fluoride-containing mouthrinses, avoiding immediate toothbrushing after vomiting, and reinforcing oral hygiene practices (Chakraborty and Anjankar, 2022[3]).

Despite the worldwide expansion of semaglutide and tirzepatide use, evidence on their direct implications for oral health remains scarce. To date, no studies have specifically associated tirzepatide directly with oral complications; however, gastrointestinal adverse effects related to this class of drugs may compromise oral health, as previously reported (Rodriguez et al., 2024[5]). This gap highlights the urgent need for further research to clarify their clinical impact on the oral cavity and to establish evidence-based preventive and management strategies for affected patients.

Semaglutide and tirzepatide represent significant advances in the management of metabolic disorders and weight reduction. However, their increasing use for aesthetic purposes raises concerns regarding both systemic and oral complications (Ryan et al., 2024[6]). Adverse effects such as xerostomia, dysgeusia, halitosis, dental erosion, and hypersensitivity highlight the importance of vigilance among dental practitioners (Bando et al., 2024[2]; Mawardi et al., 2023[4]; Volpe et al., 2023[10]). These oral alterations are summarized in the Supplementary information (Supplementary Figure 1). Given the growing number of individuals undergoing these therapies, it is essential that dental professionals recognize such potential manifestations and closely monitor affected patients. Early identification and appropriate management of these oral complications can play a pivotal role in maintaining oral health and improving overall quality of life. Enhanced clinical awareness and further research are needed to clarify the underlying mechanisms and minimize risks associated with these treatments.

## Declaration

### Acknowledgments

None.

### Conflict of interest

The authors declare no conflict of interest.

### Artificial Intelligence (AI) - assisted technology

The authors declare that they have not used AI in the writing and production of the present manuscript.

## Supplementary Material

Supplementary information
