# Supplementary material for: Semaglutide and tirzepatide: Oral cavity effects of weight-loss therapies
Source: EXCLI J. 2026 Jan 9;25:167–9. doi: 10.17179/excli2025-9065 (PMC12946442; doi:10.17179/excli2025-9065)
Supplement: Supplementary information [file EXCLI-25-167-s-001.pdf]

EXCLI Journal 2026;25:167-169 – ISSN 1611-2156
Received: October 30, 2025, accepted: November 21, 2025, published: January 09, 2026

Supplementary information to:

Letter to the editor:

SEMAGLUTIDE AND TIRZEPATIDE: ORAL CAVITY EFFECTS OF WEIGHT-LOSS THERAPIES

Maria Eduarda Bernardo[a], Felipe Gomes Dallepiane[a*], Mario Escobar[a,c], Ariadne Cristiane Cabral Cruz[a,b], Cesar Augusto Magalhães Benfatti[a]

[a] Post-Graduation Program of Dentistry, Center for Education and Research on Dental Implants, Federal University of Santa Catarina, Florianópolis, Brazil
[b] Applied Virology Laboratory, Federal University of Santa Catarina, Florianópolis, Brazil
[c] Department of Dentistry, Universidad Católica Santiago de Guayaquil (UCSG), Guayaquil, Ecuador

* **Corresponding author:** Felipe Gomes Dallepiane, Department of Dentistry, Center for Education and Research on Dental Implants (CEPID), Federal University of Santa Catarina (UFSC), 88040-900 Florianópolis, Santa Catarina, Brazil;
E-mail: dallepianefe@gmail.com

https://dx.doi.org/10.17179/excli2025-9065

This is an Open Access article distributed under the terms of the Creative Commons Attribution License (https://creativecommons.org/licenses/by/4.0/).

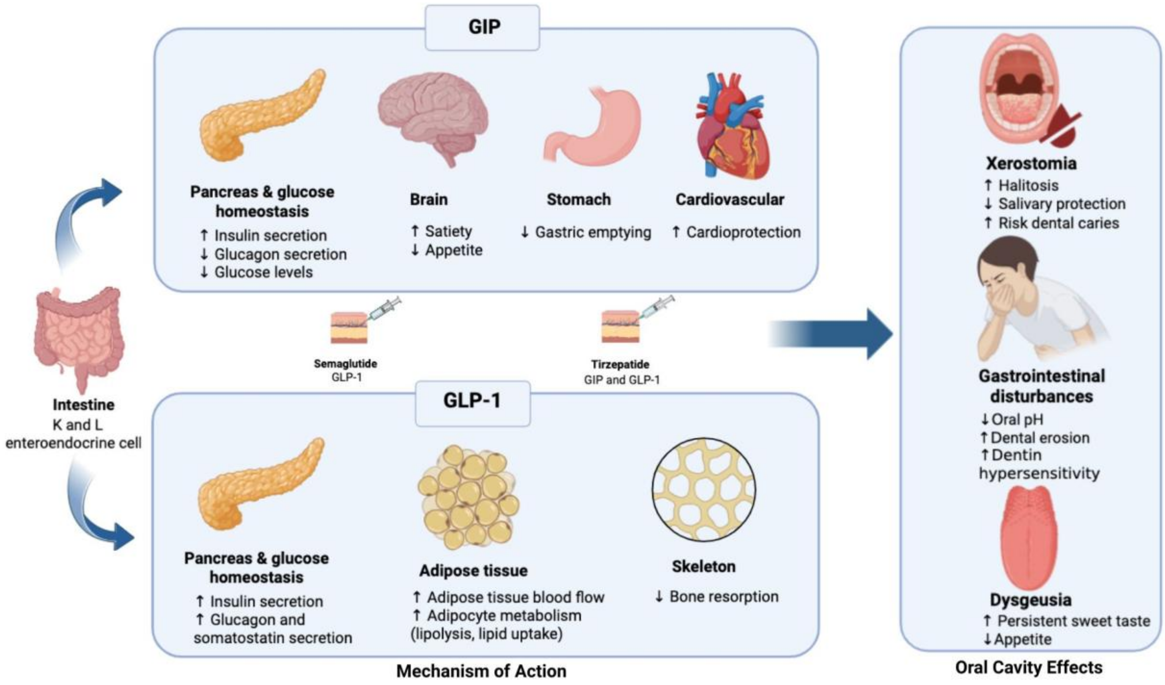

Supplementary Figure 1: Oral cavity effects associated with semaglutide and tirzepatide use
